# Comparison lecture and concept map methods on the level of learning and satisfaction in puerperal sepsis education of midwifery students: a quasi-experimental study

**DOI:** 10.1186/s12909-023-04247-8

**Published:** 2023-04-17

**Authors:** Azita Yarmohammadi, Farideh Mostafazadeh, Samira Shahbazzadegan

**Affiliations:** 1 Department of Midwifery, School of Nursing and Midwifery, Ardabil University of Medical Sciences, Ardabil, Iran; 2 Present Address: Department of Educational Technology, Faculty of Psychology and Education, Allameh Tabataba’i University, Tehran, Iran

**Keywords:** Puerperal sepsis, Concept map, Lecture, Kirkpatrick assessment, Midwifery

## Abstract

**Background:**

Education and training about emergency cases are necessary for different medical groups such as midwives. Teaching puerperal sepsis is important for midwives. The teaching method is one of the challenges of the educational system in universities. This study was conducted to compare lecture and concept map methods on the level of learning and satisfaction in puerperal sepsis education of midwifery students.

**Method:**

This semi-experimental study was conducted in 2022 at Ardabil Nursing and Midwifery School on 50 midwifery students. Students randomly were placed in lecture and concept map teaching groups. To collect data, a 23-question satisfaction questionnaire and a 15-question test taken from the WHO books on the management of puerperal sepsis were used to check students' knowledge and learning. The data were analyzed by using descriptive statistics and independent and paired t-test SPSS software.

**Findings:**

The average learning score of the students after teaching in the concept map group was 10.28 ± 1.90 and the lecture group 9.20 ± 1.70, the difference was statistically significant (*p* = 0.04). The average satisfaction score in the concept map group was 107.92 ± 4.46 and in the lecture group 105.68 ± 6.84, this difference was statistically significant (*p* = 0.03).

**Conclusion:**

The teaching of puerperal sepsis with the concept map method had a greater effect on the learning and satisfaction of midwifery students. Therefore, it is recommended to use this educational method.

## Background

In the field of midwifery education, training and evaluation of emergency cases have a special place. Postpartum infection is the third cause of maternal mortality, especially in developing countries. Since postpartum infection is responsible for 11 mothers' deaths per 1,000 live births in 2021 and accounts for 15% of all maternal deaths in developing countries, midwives must have the ability to prevent and promptly manage this disease [1].

Unfortunately, today, a significant gap has been reported between the current performance of nurses and midwives and their training [2]. Midwives and nurses play a vital role in the prevention of puerperal infections, and the smallest negligence or simple neglect can turn the situation into an abnormal condition in a short period and a successful delivery can quickly turn into a disaster. Therefore meaningful and effective learning opportunities must be provided for nursing and midwifery students [3]. One of the most important challenges is choosing the teaching method, after selecting the content and before determining the teaching tool. The teaching method can determine how to conduct comprehensive activities to achieve educational goals [4]. A good teacher should choose the appropriate method according to the existing conditions to carry out the teaching process [5, 6]. In other words, the success of the learning process is always related to the teaching methods used by the teacher [6].

One of the common methods that have a long history in the educational system is the lecture method, which is still used more than other methods. In this method, the learner receives the material one way, and the teacher permits to ask questions and express a comprehensive opinion based on his ability. In this method, individual differences are not taken into account and the training conditions are the same for everyone. In terms of providing the amount of information, it is more affordable, and large amounts of content can be transferred in a short time [7].

A concept map is a graphical tool of organized information that can show the arguments of the learner and the teacher in a visual form [8] and is a geometric representation of purposeful and connecting relationships between ideas and concepts [9, 10]. This method is used to display knowledge, produce knowledge, share knowledge, and evaluate it to understand the amount of learning [11]. Therefore, meaningful learning is achieved when the learner can establish a relationship between new and previous information. From the point of view of many experts, one of the new and appropriate methods of learning is the use of a concept map, which is considered a method to achieve development in the teaching of important ideas and is formed with an all-encompassing focus on the main idea over time [12].

Kirkpatrick's four-level model is one of the best evaluation methods. Based on this model, four questions encountered in each training course, and answers of these questions in reaction, learning, behavior, and result levels were evaluated [13].

In recent years, limited studies have been conducted regarding the learning and empowerment of midwifery students about postpartum infection. In these studies, different and sometimes contradictory results were obtained from the use of the concept map method and its impact on students' learning. Laight et al. showed that students can be understood the relationship between concepts through the concept map teaching method. In this way, the concept map has an important application in creating motivation and creativity in learning and teaching adult classes [14].

Considering the importance of knowledge about causes and symptoms of postpartum infections and the training of midwives of controlling and managing this disease, the teaching of this subject is felt with an emphasis beyond what the students spend in their courses. There is no agreement on the best teaching method in emergency cases such as puerperal sepsis education. For this reason to improve midwifery students’ skills and regard to limited and contradictory results in past studies about the use of concept map method, this study was conducted with the aim of comparison lecture and concept map methods on the level of learning and satisfaction in puerperal sepsis education of midwifery students.

## Methods

### Study design and samples size

**Fig. 1:**
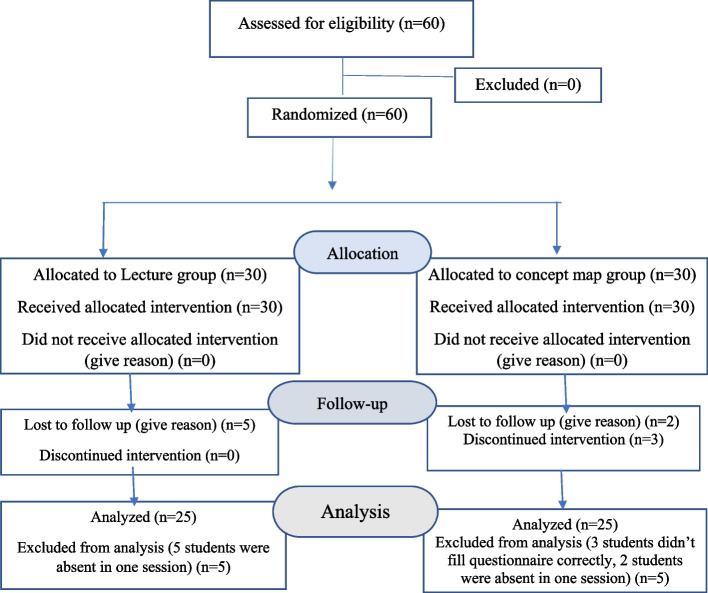
Flow diagram of recruitment and retention of participants in the study

This semi-experimental study was carried out after obtaining ethics approval: IR.ARUMS.REC.1401.053. In Ardabil Nursing and Midwifery school on Jun 2022. The research population was 60 midwifery students, who were randomly divided into two groups: A: teaching by lecture method and B: teaching by concept map method. According to the names in the class list, students with odd numbers were placed in the lecture group and students with even numbers were placed in the concept map group. The inclusion criteria were passed the first pregnancy and delivery unit and have a previous background regarding the stages of childbirth. The exclusion criteria were absence in at least one session and failure to fill in the questionnaire correctly. 10 students were excluded from the study due to defects in filling out the questionnaire (3 people) and non-attendance in one session (7 people). Finally, 50 students were completed the study (Fig. 1). The sample size was calculated about 26 students for each study group using G Power software whit α═0.05, power═ 0.85, effect size═ 0.70 and PASS Software with α═0.05, power═ 0.96, SD = 2, effect size = 0.5.

First, a briefing session was held regarding the objectives of the research and the request for cooperation in the implementation of the research, emphasizing accuracy and honesty in completing the questionnaires and requested information. The participants were assured that the obtained information would be completely confidential. Before teaching, the pre-test was taken. The training was first conducted in the lecture group and then the teaching of the concept map group was started instantly to prevent information sharing between the two groups. The training was conducted by the researcher in two sessions for 3 h for both groups in two consecutive weeks. At the end of the training sessions, a satisfaction questionary was completed by the participants. After two weeks, the post-test was held again for the participants in both groups, post-test questions were considered the same as the pre-test.

### Intervention

In the lecture group, teaching in the common way of universities and question and answer along with PowerPoint was presented for six hours in two sessions of three hours for two weeks. The educational content presented in the first session included: the introduction and definition of puerperal sepsis, pathogenic factors and risk factors of puerperal sepsis, avoidable factors, problem identification, and examination for the diagnosis of puerperal infection. In the second session, the educational content of management and treatment of cystitis and pyelonephritis, mastitis and peritonitis, thromboembolic disorders, and chorioamnionitis along with infection prevention procedures were taught. During teaching for the lecture group, questions and answers were conducted between the researcher and the learners as needed.

In the concept map group, at the beginning of the session, the students were introduced to the concept maps and their constituent parts and how to draw them. In this research, the goal of presenting the concept map to the teacher was that the person can find out the main information and the relationships between them with an overview, and the brain can easily interpret the concepts and their relationships. It was shown to the students by the researcher using PowerPoint software. The educational content related to the introduction and definition of puerperal sepsis, pathogenic factors and risk factors of puerperal sepsis, avoidable factors, problem identification, and examination for the diagnosis of puerperal infection was taught in the form of a concept map. In this teaching method, the educational content with concept maps was prepared by the researcher and the concept maps were available to the students. During the lesson, the students were able to draw them in their notebooks by looking at the maps. In the second session, the educational content was also the management and treatment of cystitis and pyelonephritis, mastitis and peritonitis, thromboembolic disorders, and chorioamnionitis along with the training in infection prevention procedures. Also, a history of a patient suffering from chorioamnionitis was given to the students, and the students were asked to draw a conceptual map according to the learned content, and at the end of the maps drawn by the students, they were considered the subject of behavioral exchange.

In terms of ethical considerations, obtaining approval from the ethics committee and obtaining permission to hold a workshop, the anonymity of the questionnaires, and the use of information only in the implementation of the research, and the explanation of the study method to the participants were noticed.

### Measurement

The data collection tool consisted of three parts: the first part was the personal characteristics of the learners, including age, academic semester, and marital status. The second part was to measure the knowledge of the learners in the field of different aspects of postpartum infection. This questionnaire consisted of 15 four-choice questions, which were taken from the World Health Organization (WHO) book of educational principles for midwifery educators in the field of puerperal sepsis [15]. Questions were designed according to the content of teaching in three topics: 1. Recognition and causative, 2. Avoidable factors, and 3. Identification and management of puerperal sepsis. To score the knowledge test, one score was given for correct answers, and zero scores for incorrect and no answers. The total scores ranged between zero and fifteen. To evaluate the content validity of the test, it was given to 10 experts. The content validity index was measured 0.87. For reliability and internal consistency test, questionnaire was completed by 10 midwifery students, and Cronbach's alpha coefficient was obtained 0.78. In the third part, students' satisfaction was measured based on the 23-question satisfaction questionnaire with a five-point Likert scale from very low to very high. The first eight questions were related to the content, the next nine questions were to measure the level of satisfaction with the lecturer, the next five questions were to examine the students' satisfaction with the organization and facilities of the course, and finally, the overall satisfaction with the course was examined. For the review of the content index, the way of content presentation, degree of applicability, quality of the content and its relationship with the presented content, innovation of content, promotion of professional knowledge, course objects, suitability of course objects with study time, usefulness of educational texts were taken into account. The teacher's index including ability to express and understand the material, success of the teacher in conveying the material, expertise and mastery of the teacher in presenting, appropriate use of various teaching methods, optimal use of time during teaching, teaching method, degree of student participation during the class, concluding of the session at the end of class, and the teacher's general evaluation (teaching, order, behavior, etc.) were reviewed. In the organization-facilities section, educational environment and facilities (light, heat, noise), discipline of the course, the course administration, audio and visual educational facilities, the performance and cooperation of the course officials, and the level of overall satisfaction with the course were assessed.

### Statistical analysis

To describe the personal-social characteristics of the participants and to check students' learning from descriptive statistics including absolute and percentage frequency, mean and standard deviation, to check the normality of data distribution from the Shapiro–Wilk and Kolmogorov–Smirnov tests and to compare the mean scores independent and pairs t-test, and Mann–Whitney U were used via SPSS software version 26.

## Results

In this study, 50 bachelor midwifery students were studied. Demographic information of the students including age, marital status, and term semester was shown in Table 1. The average age of the students was 22.36 ± 1.62 years with a range of 19 to 27 years. The minimum academic term was the 4th semester and the maximum was the 8th semester. 4 students were married and the rest were single. There was no significant difference in the respect of demographic characteristics between the two groups (*p* > 0.70).

**Table 1:** Demographic information in two groups of lectures and concept maps in students

Groups		Concept map (*n* = 25)	Lecture (*n* = 25)	*p-value*
Variable		Mean ± SD	Mean ± SD	
Age (year)		22.28 ± 1.64	22.44 ± 1.63	0.73*
Semester (Number (%))	4	8 (32%)	8 (32%)	0.73^+^
	6	10 (40%)	8 (32%)	
	8	7 (28%)	9 (36%)	
Marital status (Number (%))	Single	23(92%)	23(92%)	1.00^+^
	Madrid	2 (8%)	2 (8%)	

^*^Independent t-test, ^+^Mann–Whitney U

**Fig. 2:**
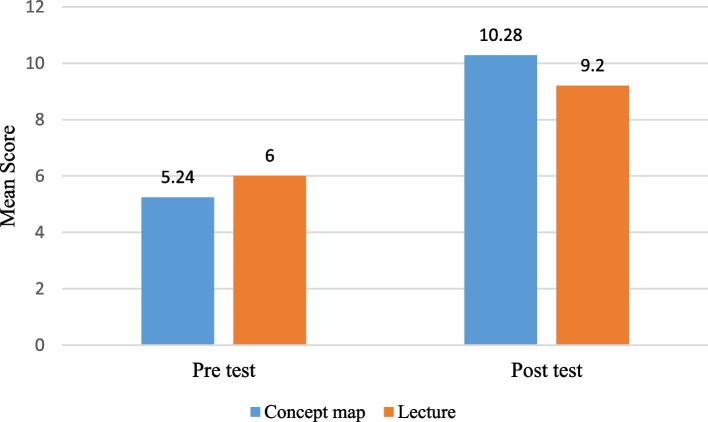
Learning mean score of students in the pre-test and post-test in two groups

Learning mean scores of students before the study were 6.00 ± 1.97 in the lecture group and 5.24 ± 2.18 in the concept map group, this difference was not significant (*p* = 0.20). These scores were increased to 9.20 ± 1.70 in the lecture group and 10.28 ± 1.90 in the concept map group after training. Paired t-test showed that these changes in learning scores were significant in both groups (*p *< 0.001) (Fig. 2). The level of reaction and learning score in the two groups were shown in Table 2. The student’s learning score averages after teaching in the concept map and lecture groups were 10.28 ± 1.90 and 9.20 ± 1.70, respectively. Their difference was statistically significant (*p* = 0.04). The average satisfaction score in the concept map and lecture groups were 107.92 ± 4.46 and 105.68 ± 6.84, respectively. The satisfaction score was statistically higher in the concept map group (*p* = 0.03).

**Table 2:** Comparison of the reaction and learning scores in two groups of lectures and concept maps in students

Groups		Concept map	Lecture	*p*-value*
		(*n* = 25)	(*n* = 25)	
First two-level of Kirkpatrick's Training Model		Mean ± SD	Mean ± SD	
Reaction	Program objectives	43.24 ± 1.66	42.16 ± 2.80	0.044
	Engagement	27.24 ± 2.12	26.56 ± 2.29	0.889
	Relevance	37.44 ± 1.80	36.96 ± 2.90	0.007
	Satisfaction	107.92 ± 4.46	105.68 ± 6.84	0.032
Learning	Pre test	5.24 ± 2.18	6.00 ± 1.97	0.204
	Post test	10.28 ± 1.90	10.28 ± 1.90	0.040

^*^Pairs t-test

## Discussion

Today, promoting meaningful learning is one of the main goals of education and an important factor in creative thinking. This research was conducted to compare the teaching of puerperal sepsis with two methods of lectures and concept maps based on the first two levels of Kirkpatrick's model of midwifery students in Ardabil at 2022. The result showed that the training group had more satisfaction and learning in the concept map method. This result is in consistence with the findings of Hanani et al. [16], Dehghanzadeh and Moaddab [17], Banerjee et al. [18], Nair et al. [19]. Hsu et al. showed that use of outcome-based on concept mapping as educational method could encourage taking of bio-psycho-social approach to medicine by nursing students and ultimately resulted in better nursing care quality [20]. According to Hanani et al. [16], Pouragha [21], and Ramasubramaniam et al. [22] concept map method is effective in increasing the recall and positive satisfaction of nursing students.

The evaluation of learning score showed that this criteria was increased after intervention in both groups. This result is in agreement with Islami et al. [23]. Rigi et al. Showed that the clinical skill of preeclampsia control in both the concept map training and the traditional method groups after two weeks of the intervention significantly increased compared to before the intervention [24]. Hasanvand et al. reported that average score of the total creativity and learning and all scales in the two groups decreased after intervention, which is in opposition to our study. They attributed this decrease to the use of the conceptual map model in English and most of the participants did not master in English [25].

Dehghanzadeh and Moaddab also concluded that the concept map method capable to strengthening learning in the areas of analysis and inductive reasoning, critical thinking, and care program design in nursing students. Their research showed that the critical thinking scores of the experimental group in the areas of analysis, comparative reasoning, and nursing process scores were significantly higher than the control group [17]. Fatawi et al. showed that the concept map not only improves the learning results but also increases the student's participation in all types of studies, i.e. behavioral, emotional, and cognitive [26]. Kofi et al. showed the average genetic scores during the post-test was better in concept map method group compare to control group [27]. Utami and Yuliyanto stated that there was a significant difference in terms of learning results in students before and after learning using the concept map model and strategies are effective in students' motivation learning [28]. Silva et al. found that concept maps improved further critical and creative thinking skills in relation to the lecturing class [29]. Karimi mentioned the usual educational program in the control group could not improve students' critical thinking skills and academic progress, and that traditional educational methods for cultivating critical thinking need to be reformed [30]. Piri et al. showed teaching with the concept map method can increase the level of memory of students [31].

The findings of the present research showed that the use of concept maps provided high satisfaction of students from teaching puerperal infection which is in accordance with studies of Freeman et al. [32] Zaitseva et al. [33], Hanani et al. [16]. Elsayed Farid et al. showed that 86% of the students in the electronic mind-mapping group were satisfied, while only 55% in the conventional group were satisfied [34].

The strength of the study was designing. Limitations of this research were the impossibility of examining the research on the third and fourth levels (Behavior and Results) of Kirkpatrick's evaluation due to time limitations and blinding of the study.

## Conclusion

The findings of the research showed the superiority of the concept map method compared to the lecture method on the level of satisfaction and learning in teaching of puerperal infection of midwifery students. Promotion of midwifery student’s education will help to the mother, child and society’s health. Use of concept map method is recommended for teaching of other important subjects in the field of midwifery.

## Data Availability

The datasets generated during and analyzed during the current study are available from the corresponding author.

## Abbreviation

CM: Concept Map
